# Complete Chloroplast Genome Sequence of an Orchid Model Plant Candidate: *Erycina pusilla* Apply in Tropical *Oncidium* Breeding

**DOI:** 10.1371/journal.pone.0034738

**Published:** 2012-04-04

**Authors:** I-Chun Pan, Der-Chih Liao, Fu-Huei Wu, Henry Daniell, Nameirakpam Dolendro Singh, Chen Chang, Ming-Che Shih, Ming-Tsair Chan, Choun-Sea Lin

**Affiliations:** 1 Institute of Biotechnology, National Cheng Kung University, Tainan, Taiwan; 2 Agricultural Biotechnology Research Center, Academia Sinica, Taipei, Taiwan; 3 Academia Sinica Biotechnology Center in Southern Taiwan, Tainan, Taiwan; 4 Department of Molecular Biology and Microbiology, University of Central Florida, Orlando, Florida, United States of America; 5 Department of Horticulture, National Chung Hsing University, Taichung, Taiwan; National Taiwan University, Taiwan

## Abstract

*Oncidium* is an important ornamental plant but the study of its functional genomics is difficult. *Erycina pusilla* is a fast-growing Oncidiinae species. Several characteristics including low chromosome number, small genome size, short growth period, and its ability to complete its life cycle *in vitro* make *E. pusilla* a good model candidate and parent for hybridization for orchids. Although genetic information remains limited, systematic molecular analysis of its chloroplast genome might provide useful genetic information. By combining bacterial artificial chromosome (BAC) clones and next-generation sequencing (NGS), the chloroplast (cp) genome of *E. pusilla* was sequenced accurately, efficiently and economically. The cp genome of *E. pusilla* shares 89 and 84% similarity with *Oncidium* Gower Ramsey and *Phalanopsis aphrodite*, respectively. Comparing these 3 cp genomes, 5 regions have been identified as showing diversity. Using PCR analysis of 19 species belonging to the Epidendroideae subfamily, a conserved deletion was found in the *rps15-trnN* region of the Cymbidieae tribe. Because commercial *Oncidium* varieties in Taiwan are limited, identification of potential parents using molecular breeding method has become very important. To demonstrate the relationship between taxonomic position and hybrid compatibility of *E. pusilla*, 4 DNA regions of 36 tropically adapted Oncidiinae varieties have been analyzed. The results indicated that *trnF*-*ndhJ* and *trnH*-*psbA* were suitable for phylogenetic analysis. *E. pusilla* proved to be phylogenetically closer to *Rodriguezia* and *Tolumnia* than *Oncidium*, despite its similar floral appearance to *Oncidium*. These results indicate the hybrid compatibility of *E. pusilla*, its cp genome providing important information for *Oncidium* breeding.

## Introduction

The Orchidaceae has great diversity in floral morphology and a rich array of species. It comprises the largest family of flowering plants [1]. The extraordinary variety of orchid floral features and appearances ensure a large consumer demand for orchids. *Oncidium*, a genus in subtribe Oncidiinae, is a popular and important cut flower. It needs about 3 years to reach sexual maturity under natural conditions [2], its flowering being precisely regulated by temperature [3]. Taiwan, located in tropical and subtropical areas with higher temperature, is one of the *Oncidium* cultivation and hybridization center of world [4]. To develop new commercial species with competitive advantageous traits such as shorter vegetative stage or tropical growth of *Oncidium* species continues to be a pressing need.

For orchid breeding, it is important for molecular studies of orchid to help in efforts to create unique flower colors and shapes, as well as disease-resistant cultivars that are of high economic value. In Taiwan, genomics approach by transcriptomic database establishment [5]–[7] chloroplast genome [8], [9], and transformation technology have been applied in orchid research [10], [11]. Few transgenic orchids have been obtained and used in orchid breeding [12]. Sweet pepper ferredoxin-like protein (*pflp*), a new selection marker developed by You et al., was over-expressing in *Oncidium* orchid “Sherry Baby cultivar OM8” and enanced *Erwinia carotovora* resistance of transgenic orchid [13]. Transgenic *Phalaenopsis* expressing coat protein of *Cymbidium* mosaic virus (CymMV) enhanced protection against CymMV infection through RNA-mediated resistance [14]. However, several disadvantages that make orchid breeding by either traditional hybridization or gene engineering difficult are: (1) most of the plants grow slowly; (2) there is a wide range of chromosome numbers e.g. *n* = 6–30 in Oncidiinae [15]; (3) genome sizes are large and have complex polyploidy caused by spontaneous or man-made hybridization [16]–[18]. Many *Oncidium* genes have been cloned and studied by ectopic expression in *Arabidopsis* or *Eustoma* [19]–[21], however, gain-of-function studies in orchid remains scarce. Therefore, an orchid model plant system is needed for the functional genetic investigation.

*Erycina pussila*, is a fast-growing epiphytic orchid with a relatively low chromosome number (*n* = 6; [22]) and small genome size (1.5 pg per 1C nucleus; [23]). Pollination and production of seed capsules rarely occurs in nature [24]. Currently, advances in cultural techniques and precocious flowering have meant that *E. pusilla* can be grown rapidly, and will produce flowers and fruit *in vitro* [25], [26]. These characteristics make *E. pusilla* not only an attractive model plant for functional genomic and flowering studies of *Oncidium*, but also an excellent parent for traditional hybridization methods. To produce attractive traits and breed new commercial orchid species, *E. pusilla* has been crossed with several important Oncidiinae orchids, and different hybridization compatibility was found with *Oncidium*, *Rodriguezia* and *Tolumnia* [27]. However, systematic molecular investigations and genomic information on *E. pusilla* remain unclear.

Chloroplast DNA is useful in evolutionary studies because of its simple structure, highly conserved sequence, and maternal inheritance characters [28]. Several plastid regions, such as *matK*, *atpB*, *psbB*, *psbC* and *rpoC1*, have been used to identify phylogenetic relationships in orchid [29], [30]. Sequencing of complete plastid genomes of different genera has recently provided useful information regarding RNA editing and loss of introns [31], [32]. Chloroplast (cp) genomes of 2 orchids, *Phalaenopsis aphrodite* and *Oncidium* Gower Ramsey, have been sequenced [8], [9]. The availability of chloroplast genome sequences should also help in developing genetic engineering including chloroplast transformation [33], [34]. Information on the complete chloroplast genome sequence is not only important for taxonomic classification but also for crop improvement.

To provide information on breeding and molecular aspects of *Oncidium*, we have sequenced the complete cp genome of *E. pusilla* using BAC library and next-generation sequencing (NGS). To demonstrate the possibility this orchid as a model, the difference between its cp genome and those of other important orchid species, such as *Phalaenopsis* and *Oncidium*, have been compared. Primers were designed based on the various regions of 3 cp genomes and 19 Epidendroideae species were analyzed by PCR. To investigate the cross compatibility of *E. pusilla* in Oncidiinae species, several cp DNA regions of 36 *Oncidium* species were obtained and applied for phylogenetic analysis.

## Materials and Methods

### Chloroplast-BAC clone identification

Young *E. pusilla* leaves (200 g) grown *in vitro* were collected for isolation of high molecular weight DNA [35]. The DNA was partially digested by *Hind*III, and the fragments ligated into vector pCC1BAC DH10b (Amplicon, Pullman, WA), which was used for transfection into *E. coli*. Individual clones were picked up and placed into 384-well plates that contained liquid LB medium with 12.5 mg/L chloramphenicol. The plates were incubated at 37°C overnight and stored at −80°C. Chloroplast specific primers designed by Wu *et al*. [9] were used to amplify predicted chloroplast regions from a BAC library. The BAC clones containing chloroplast regions of interest were obtained by PCR screening from super pool, plate, row, and spot, as described by Hsu *et al*. [10]. BAC clones of the chloroplast were identified (clone ID P-5-K16).

### Illumina sequncing

BAC plasmids for Illumina sequencing were isolated using the NucleoBond BAC 100 kit (NucleoSpin Blood kit, Macherey-Nagel, Germany). Five micrograms of *E. pusilla* BAC plasmid were sheared into fragments of 200–600 bp by Bioruptor Next Gen (Diagenode) in 100 µl TE buffer. The purified DNA fragments were treated with T4 DNA polymerase, *E. coli* DNA polymerase I Klenow fragment and T4 Polynucleotide Kinase. Adapters required for sequencing on the Illumina platform were added to DNA fragments. The ligation products were separated on a 2% agarose gel; those between 270 and 350 bp were excised, eluted from the gel slice, precipitated and resuspended in 15 µl TE, using QIAquick Gel extraction Kit (Qiagen). The adapter-modified DNA fragments were amplified, and the products purified using an Agencourt AMPure XP (Beckman). They were collected in 30 µl of QIAGEN elution buffer (Qiagen). After quantification by Quant-iT dsDNA HS Assay Kit (Invitrogen) and KAPA Library Quantification Kit (KAPABiosystem), the molar concentration was calculated and the quality examined by Expersion DNA 1K Analysis Kit (Bio-Rad). The DNA library was then prepared for sequencing.

### Bioinformatics

Sequencing was performed on an Illumina GA IIx platform, using a paired-end strategy at a read-length of 75 bases. Nucleotides with low quality scores (<3) were removed from the sequence reads, and any that had a 100% match to the cloning vector sequence or *E. coli* sequences were also removed from the subsequent assembly process. De novo assembly was conducted using CLC Genomics Workbench (CLC bio, Cambridge, MA). The gaps between the contigs were filled by PCR.

### *E. pusilla* chloroplast genome annotation

The cp genome was annotated using Dual Organellar GenoMe Annotator (DOGMA) [36]. This program uses a FASTA-formatted input file of the complete genomic sequences and identifies putative protein-coding genes by performing BLASTX searches against a custom database of published cp genomes. Both tRNAs and rRNAs were identified by BLASTN searches against the same database of cp genomes. For genes with low sequence identity, manual annotation was performed after identifying the positions of the start and stop codons, as well as the translated amino acid sequence, using the chloroplast/bacterial genetic code. The annotated genome sequences were submitted to NCBI (Accession no: JF_746994).

### Plant materials

Orchids were obtained and collected from a local grower in Taiwan. All orchids were maintained in the greenhouse at National Chung Hsing University, Taichung, Taiwan.

### DNA purification and genomic PCR

For chloroplast genomic PCR analysis, total genomic DNA was isolated from leaves using a urea extraction buffer system [37]. The primer designs for Epidendroideae species analysis were based on the various regions of 3 orchid cp genomes. The primer sequences, sequence sizes, and forward primer position in *E. pusilla* are shown in Table 1. Primers designed by Wu *et al*. [9] were used for Oncidiinae variety analysis (the sequences are shown in Table 1). Genomic PCR was conducted in a final volume of 50 µl containing 2.5 units of Taq DNA polymerase (Violet gene, Taipei, Taiwan), 1.25 mM of each dNTP, and 10 pmol of each primer. The amplification program used was 30 cycles at 94°C for 30 s, 55°C for 30 s, and 72°C for 90 s. The PCR products were sequenced and assembled using VectorNTI Contig Express software.

**Table 1:** Primers for Epidendroideae genes and Oncidiinae phylogenetic analysis.

	Sequence	Sequence	Position	bp
*atpH*-*atpI*	GTCTCGCAATACCTTCTACGGC	GGAGAGGAGTATGGTCCTTGGG	13740	1204
*petN*-*psbM*	GGGCTGCTTTAATGGTAGTGTTTAC	TTATTGCTACTGCGCTGTTCATTT	28958	933
*accD*-*psaI*	GCTGAACCTAATGCCTACATTGC	AGAAGCCATTGCGATTGCC	58330	928
*psbE*-*petL*	CAACCCGCAATGAATAGGGA	GGGTTATAGTTGAAGCAGCCAGTA	64478	946
*rps15*-*trnN*	CTTTGAATCGAAGATATAGCAATTTCC	AAAAAACGGGGACCAAGAAATT	114398	762
*trnH*-*psbA*	AAGCGTCCTGTAGTAAGAGGA	GGGAAACCACTGAAAATGAG	142236	1413
*matK*	TCTAGCACACGAAAGTCGAAGT	CGATCTATTCATTCAATATTTC	1875	936
*trnF*-*ndhJ*	TCGGGATAGCTCAGTTGGTA	GTTTCTGCTTCACGAATATG	49294	946
IRb-SSC	AAAATCTTCGTAAACCGGGC	ATTCGAACCTACGACCAGTCA	118168	1390

Primer sequences, annealing position of the forward primer in *E. pusilla*, and the PCR amplification length are presented. The first 5 sets of primer were used for Epidendroideae analysis. The last 4 sets of primer were used for Oncidiinae phylogenetic analysis [9].

### Analysis of sequence variability of 3 orchid cp genomes and Epidendroideae species

Chloroplast sequences of *E. pusilla*, *Onc*. Gower Ramsey (GeneBank accession NC_014056), and *P. aphrodite* (GeneBank accession NC_007499) were used for genome comparison. For Epidendroideae species analysis, 5 intergene regions (*atpH*-*atpI*, *petN*-*psbM*, *accD*-*psaI*, *psbE*-*petL*, and *rps15*-*trnN*) were obtained by PCR from 19 varieties (Accession no:JN638455–JN638514). Sequences were compared and adjusted using the VectorNTI AlignX software program (vers. 7.0; Invitrogen, Carlsbad, CA; parameters: overlap: 30; identity: 0.95; cutoff score: 40).

### Phylogenetic analysis of Oncidiinae species

Four cpDNA regions (*trnH*-*psbA*, *matK*, *trnF*-*ndhJ*, and IRb-SSC) of 36 Oncidiinae varieties were obtained by PCR (Accession no: JN598910–JN598996), and from the NCBI database (GQ915119–915130, GU132947–132991, GU136251–136287, GU175342–175358). Alignment of nucleotide sequence was performed using the Clustal X program [38] and adjusted by GeneDoc software. Phylogenetic analysis was conducted using MEGA3.1 [39], and the phylogenetic tree generated using the neighbor-joining method with 1,000 bootstrap trials by means of the neighbor-joining algorithm. Percentages of bootstrap values are indicated on the tree.

## Results

### Comparison of *E. pusilla* chloroplast genome with genomes of 2 other orchid genera

**Figure 1:**
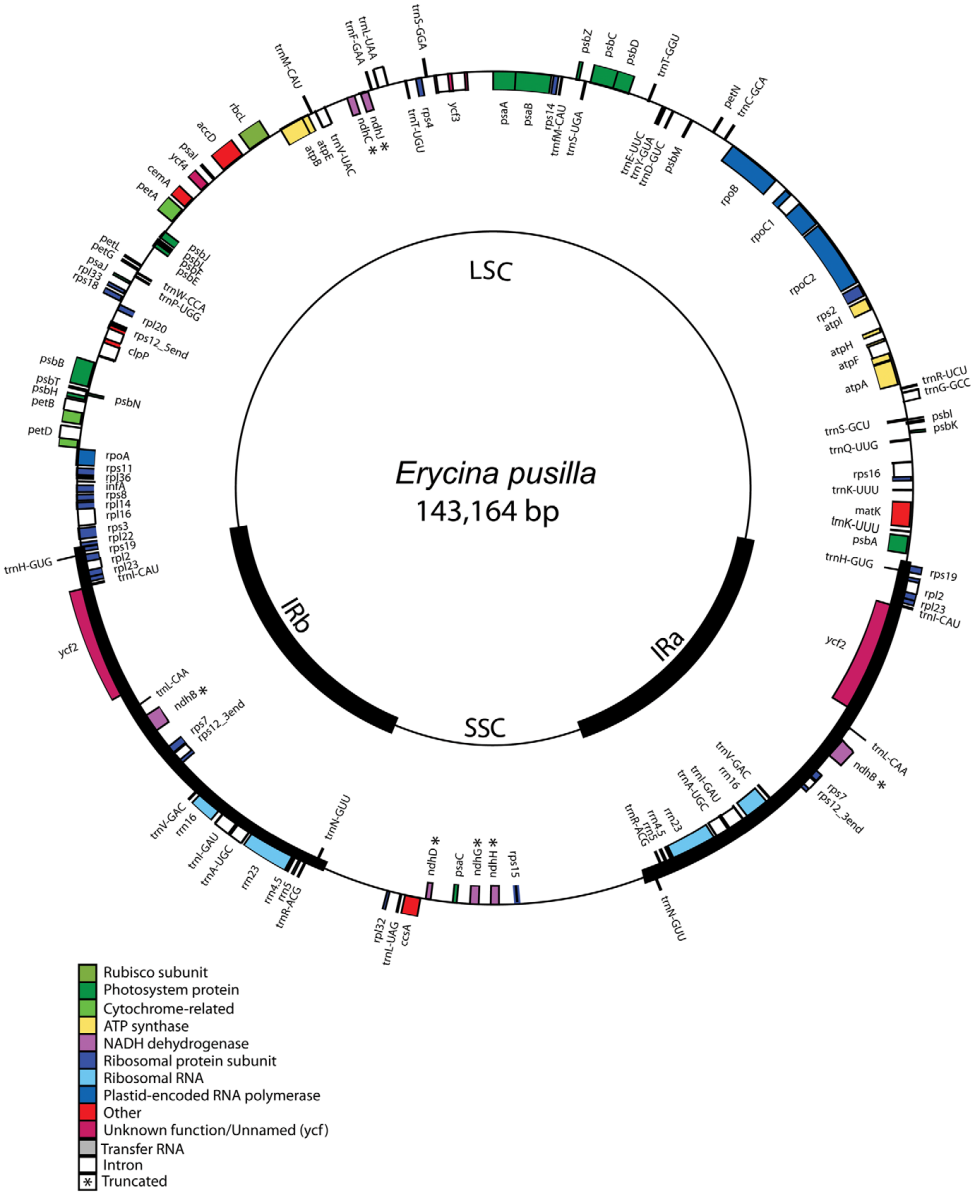
Gene map of *Erycina pusilla* chloroplast genome. Genes on the outside of the map are transcribed clockwise whereas genes on the inside of the map are transcribed counterclockwise. Colors indicate genes with different functional groups.

The cp genome of *E. pusilla* is 143,164 bp in size and contains a pair of inverted repeats (IRa and IRb) of 23,439 bp separated by large and small single copy (LSC and SSC) regions of 84,189 and 12,097 bp, respectively (Figure 1). This genome contains 126 different genes that include 73 protein coding genes, 6 pseudogenes and 19 genes duplicated in the IR region. There are 28 distinct tRNAs and 4 distinct rRNA genes. Fifteen genes contain 1 or 2 introns, and 5 of their introns are within tRNAs. The genome consists of 45.02% protein-coding genes, 46.73% non-coding DNA, which includes the intergenic spacer (IGS) regions, regulatory sequences and introns, 1.94% tRNA and 6.31% rRNA genes. The overall GC and AT content of the cp genome is 36.65% and 63.35%, respectively. The AT content of the LSC and SSC regions is 66.15% and 77.54%, respectively, whereas that of the IR region is 65.21%, including the rRNA gene cluster. The gene order of *E. pusilla* cp genome is very similar to that in the *Oncidium* cp genome.

**Figure 2:**
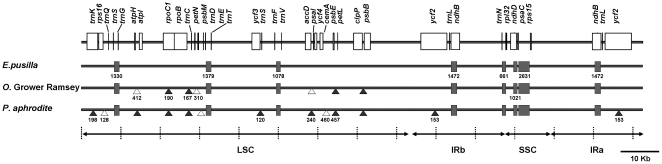
Comparison of chloroplast genomes of *E. pusilla*, *Onc*. Gower Ramsey, and *P. Aphrodite*. Deletions or insertions longer than 70 bp in *P. aphrodite* or *Onc*. Gower Ramsey in comparison with *E. pusilla* are labeled as white triangles or black triangles individually. Highly diverse sequence regions larger than 500 bp are labeled with black blocks. Numbers indicate the longest length of comparative deletions, insertions, or diverse sequence retions of three species orchids.

To understand the variation in orchid cp genomes, the *E. pusilla* cp genome was compared with the previously published *Onc*. Gower Ramsey and *P. aphrodite* genomes. The cp genome of *E. pusilla* has 89 and 84% identity with *Onc*. Gower Ramsy and *P. aphrodite*, respectively. Most insertions except *NADH dehydrogenase* (*ndh*) genes, deletions or diverse sequence regions occur within intergenic regions (Figure 2). For example, a 198 bp insertion was found in the *trnK* intron of *P. aphrodite*. The *rpoC1* introns in both *Onc*. Gower Ramsey and *P. aphrodite* were 190 bp longer than in *E. pusilla*. A 153 bp insertion was found in *ycf2* of *P. aphrodite*, making it 51 amino acids longer than in *E. pusilla* and *Onc*. Gower Ramsey. Based on the various regions of 3 orchid cp genomes, the primer for further analysis was designed (Table 1).

**Figure 3:**
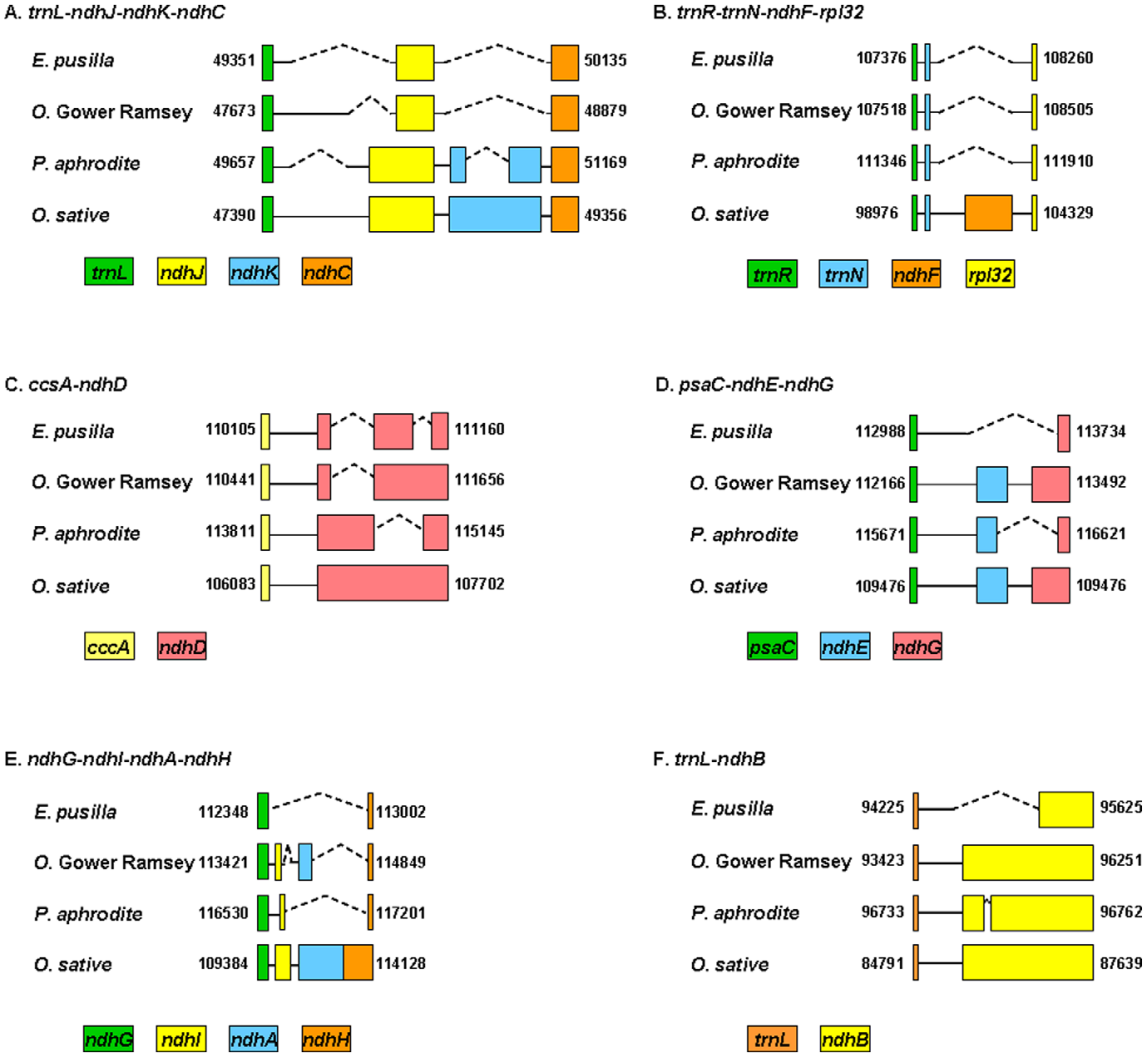
Structure of *ndh* genes in 3 orchid cp genomes. Numbers indicate the position in the chloroplast genome. The angled dashed lines indicate the gaps. Different colors indicate different *ndh* genes, a color key is shown at the bottom of each part of the Figure. Accession number of *O. sative* Japonica is NC_001320.

Among 3 orchid chloroplast genomes, sequences of ndh genes were most variable. In *E. pusilla* and *O*. Gower Ramsey, *ndhJ* was trunked and *ndhK* was abscent (Figure 3A). All *ndhF* were abscent and *ndhD* were trunked in three orchid cp genomes (Figure 3B and Figure 3C). The ndhE was only absent in *E. pusilla* (Figure 3D). The *ndhA* gene sequence was only present in *O*. Gower Ramsey (Figure 3E). The *ndhB* that located in the IR region was 892 bp in *E. pusilla*, which is 1333 and 1137 bp shorter than in *Onc*. Gower Ramsey and *P. aphrodite*, respectively (Figure 3F). These results indicate that deletion and truncation are common in chloroplast-encoded *ndh* genes of orchid plants.

### Analysis of 5 regions in 19 Epidendroideae species

**Figure 4:**
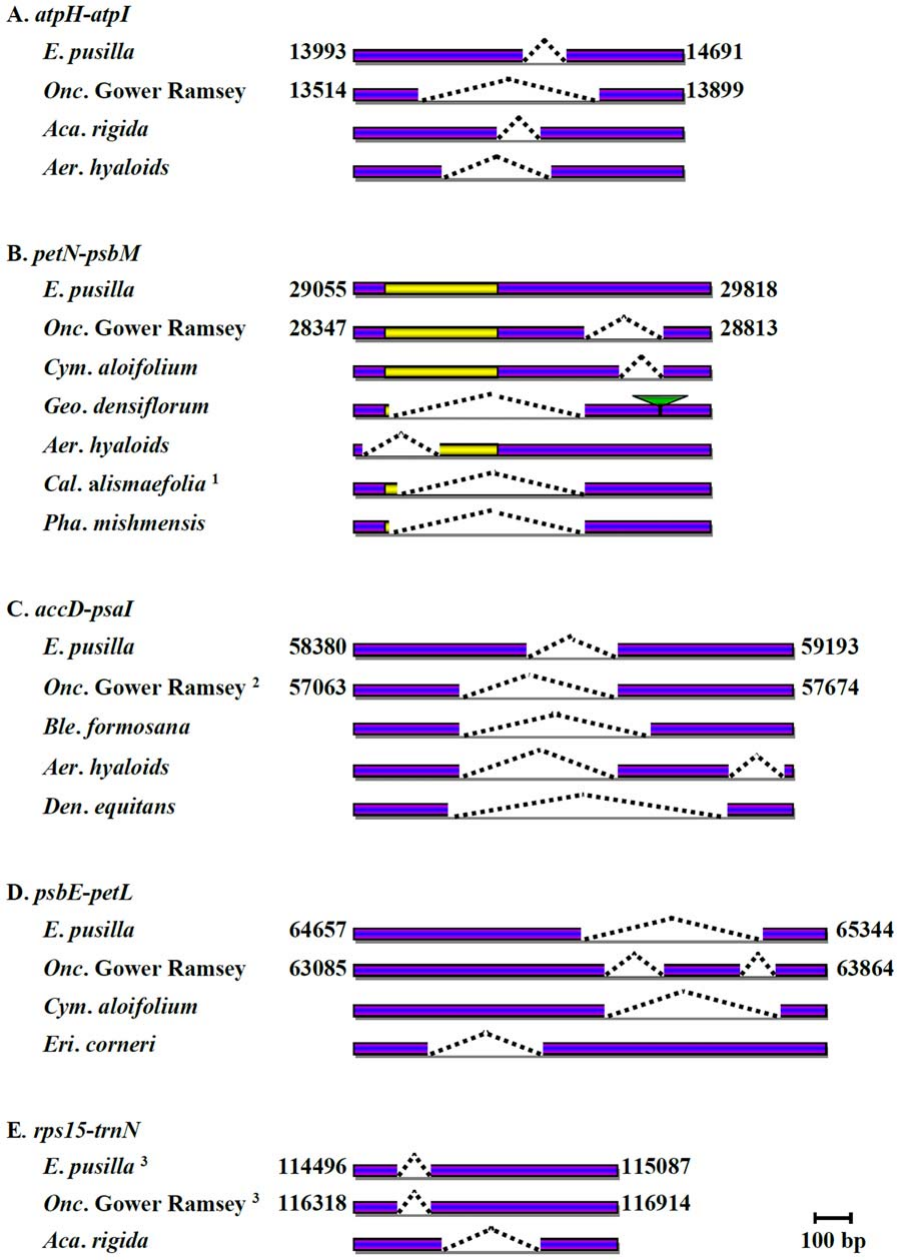
Variations in 5 regions in 19 Epidendroideae species. Numbers indicate the positions of the chloroplast genome. The angled dashed lines indicate deletions. The green triangle indicates an insertion. The yellow areas indicate diverse sequences. ^1^including *Calanthe discolor* and *Calanthe sylvatica*; ^2^including *Geodorum densiflorum*; ^3^including *Cymbidium aloifolium* and *Geodorum densiflorum*.

To analyze the genome variation in orchids, 5 regions were chosen for PCR analysis in 19 species of the Epidendroideae subfamily (Table 2). In the *atpH*-*atpI* region, 4 different deletions were found in *E. pusilla*, *Onc*. Gower Ramsey, *Acampe rigida* and *Aerangis hyaloids*, which varied in size and location (Figure 4A). The first 400 bp was highly diverse in the *petN*-*psbM* region. Three *Calanthe* species shared the same deletion. A similar but 20 bp longer deletion was found at the same position in *Geodorum densiflorum* and *Phaius mishmensis*. *Geo*. *densiflorum* contained one more insertion (Table 2 and Figure 4B). Three deletions at different locations of the *petN*-*psbM* region were found in *Onc*. Gower Ramsey, *Cymbidium aloifolium* and *Aer*. *Hyaloids* (Table 2, and Figure 4B). In the *accD*-*psaI* region, *Onc*. Gower Ramsey and *Geo*. *densiflorum* shared the same 536 bp deletion. *Aer*. *Hyaloids*, which belongs to the Angraecinae subtribe of Vandeae, contained 2 deletions (Table 2, and Figure 4C). *E. pusilla*, *Bletilla formosana and Dendrobium equitans* each contained one unique deletion (Table 2, and Figure 4C). Analysis of the *psbE*-*petL* intergene sequences showed 5 different deletions located in a disorderly fashion within *E. pusilla*, *Onc*. Gower Ramsey, *Cym*. *aloifolium* and *Eria corneri* (Table 2, and Figure 4D). In the *rps15*-*trnN* region, a deletion found in all 4 Cymbidieae species, including *E. pusilla*, *Onc*. Gower Ramsey, *Cym*. *aloifolium* and *Geo*. *densiflorum* (Table 2, and Figure 4E). With the exception of *Aca*. *rigida*, which contained another 295 bp deletion, the other species all shared similar *rps15*-*trnN* sequences (Table 2, and Figure 4E).

**Table 2:** Summary of gene patterns in Epidendroideae subfamily.

Tribe/Subtribe/Species			*atpH*-*atpI*	*petN*-*psbM*	*accD*-*psaI*	*psbE*-*petL*	*rps15*-*trnN*
**Cymbidieae**							
	**Oncidiinae**						
		*Erycina pusilla*	**•**	**○**	**•**	**•**	**▴**
		*Onc*. Gower Ramsey	**•**	**•**	**▴**	**••**	**▴**
	**Cyrtopodiinae**						
		*Cymbidium aloifolium*	**○**	**•**	**—**	**•**	**▴**
	**Eulophiinae**						
		*Geodorum densiflorum*	**—**	**△★**	**▴**	**—**	**▴**
**Arethuseae**							
	**Arethusinae**						
		*Arundina graminifolia*	**—**	**○**	**—**	**—**	**—**
	**Coelogyninae**						
		*Bletilla formosana*	**○**	**—**	**•**	**—**	**—**
**Podochileae**							
	**Eriinae**						
		*Eria corneri*	**○**	**—**	**○**	**•**	**○**
**Vandeae**							
	**Aeridinae**						
		*Acampe rigida*	**•**	**○**	**○**	**○**	**•**
		*Doritis pulcherrima*	**—**	**—**	**○**	**○**	**—**
		*Phalaenopsis aphrodite*	**○**	**○**	**○**	**○**	**○**
	**Angraecinae**						
		*Aerangis hyaloids*	**•**	**•**	**••**	**○**	**—**
**Unplaced subtribes**							
	**Collabiinae**						
		*Spathoglottis plicata*	**○**	**—**	**○**	**—**	**○**
		*Calanthe rosea*	**○**	**—**	**○**	**○**	**○**
		*Calanthe alismaefolia*	**○**	**▴**	**○**	**○**	**○**
		*Calanthe discolor*	**○**	**▴**	**○**	**○**	**○**
		*Calanthe sylvatica*	**○**	**▴**	**○**	**○**	**—**
		*Phaius mishmensis*	**—**	**△**	**○**	**○**	**○**
		*Phaius takeoi*	**○**	**○**	**○**	**○**	**○**
	**Dendrobiinae**						
		*Dendrobium equitans*	**○**	**○**	**•**	**○**	**○**

Within each detected region, different species that share the same sequences are labeled with white circles; unique deletions are labeled as black circles. The same deletions found in different species are labeled as triangles of the same color. Black stars indicate insertions. ‘—’ indicates that no PCR product was obtained.

### Phylogenetic analysis of 36 Oncidiinae species

To investigate the relationship between the molecular study and orchid breeding, 36 important *Oncidium* species were suitable for phylogenetic analysis (Table 3). Based on the variation in the 3 orchid cp genomes (Figure 2) and previous studies [9], 4 primers (Table 1) were chosen for PCR amplification and phylogenetic analysis. Using the *matK* gene, which is in a highly conserved region, *Miltassia* (*Mtssa*) was grouped with *Beallara* (*Bllra*), and *Zelemnia* (*Zlm*) was grouped with *Tolumnia* (*Tol*). Other species could be grouped by genus, but species could not be separated. *Erycina* was phylogenetically close to *Tolumnia* and *Zelenkocidium*, but distant from *Oncidium* and *Odontocidium* (data no shown). The primers for amplifying the variable regions, IRb-SSC, could only partially divide the *Oncidium* genus from the others, which was not suitable for this analysis.

**Table 3:** Parents of 36 varieties of Oncidiinae.

Genus	Variety	Ovary Parent	Pollen Parent
*Ada*	*keiliana*	*Ada keiliana*	*Ada keiliana*
*Beallara*	Eurostar	*Beallara* Tahoma Glacier	*Oncidium schrodederianum*
	Marfitch ‘Howard Dream’	*Miltassia* Charles	*Odontioda* Fremar
	Peggy Ruth Carpenter ‘Morning Joy’	*Beallara* Tahoma Glacier	*Miltonidum* Purple Queen
	Smile Eri	*Beallara* Tahoma Glacier	*Odontioda* (Toroma X Ingera)
	Tahoma Glacier ‘Sugar Sweet’	*Bratonia* Cartagena	*Oncidium* Alaskan Sunset
*Comparettia*	*ignea*	*Comparettia ignea*	*Comparettia ignea*
	*macroplectron*	*Comparettia macroplectron*	*Comparettia macroplectron*
*Degarmoara*	Flying High	*Miltassia* Jet setter	*Odontoglossum* McNabianum
*Erycina*	*pusilla*	*Erycina pusilla*	*Erycina pusilla*
*Huangara*	Niu Boy	*Leomesezia* Lava Burst	*Macradenia multiflora*
*Ionocidium*	Popcorn ‘Haruri’	*Gomesa flexuosa*	*Ionopsis utricularioides*
*Ionopsis*	*utricularioides*	*Ionopsis utricularioides*	*Ionopsis utricularioides*
*Macradenia*	*multiflora*	*Macradenia multiflora*	*Macradenia multiflora*
*Miltassia*	Olmec	*Brassia* Rex	*Miltonia* Minas Gerais
*Odontocidium*	Golden Gate	*Odontoglossum bictoniense*	*Odontocidium* Tiger Hambuhren
	Wildcat ‘Garfield’	*Odontocidium* Rustic Bridge	*Odontocidium* Crowborough
*Odontoglossum*	Margarete Holm	*Odontoglossum bictoniense*	*Odontoglossum* Hans koch
	Violetta von Holm	*Odontoglossum bictoniense*	*Odontoglossum* Bic-ross
*Oncidesa*	Little Dragon	*Gomesa echinata*	*Oncidium cheirophorum*
*Oncidium*	Gower Ramsey	*Oncidium* Goldiana	*Oncidium* Guinea Gold
	Grower Ramsey ‘Lemon Heart’	*Oncidium* Goldiana	*Oncidium* Guinea Gold
	Grower Ramsey ‘Sunkist’	*Oncidium* Goldiana	*Oncidium* Guinea Gold
	*ornithorhynchum*	*Oncidium ornithorhynchum*	*Oncidium ornithorhynchum*
	Sharry Baby ‘Tricolor’	*Oncidium* Jamie Sutton	*Oncidium* Honolulu
	Sweet Sugar Million ‘Coin’	*Oncidium* Aloha	*Oncidium varicosum*
	Tsiku Marguerite ‘Romantic Fantasy’	*Oncidium* Twinkle	*Oncidium sotoanum*
	Twinkle	*Oncidium cheirophorum*	*Oncidium sotoanum*
*Rodriguezia*	*lanceolata*	*Rodriguezia lanceolata*	*Rodriguezia lanceolata*
	*venusta*	*Rodriguezia venusta*	*Rodriguezia venusta*
*Tolumnia*	*calochila*	*Tolumnia calochila*	*Tolumnia calochila*
	Fire Ring	**—**	**—**
	Genting Angel	*Tolumnia* Irene Gleason	*Tolumnia* Linda
	Jairak Firm ‘Brown White’	**—**	**—**
*Zelemnia*	*midas*	*Zelemnia midas*	*Zelemnia midas*
*Zelenkocidium*	Little Angel	*Zelenkoa onusta*	*Oncidium flexuosum*

‘—’ indicates that no parents information were obtained.

**Figure 5:**
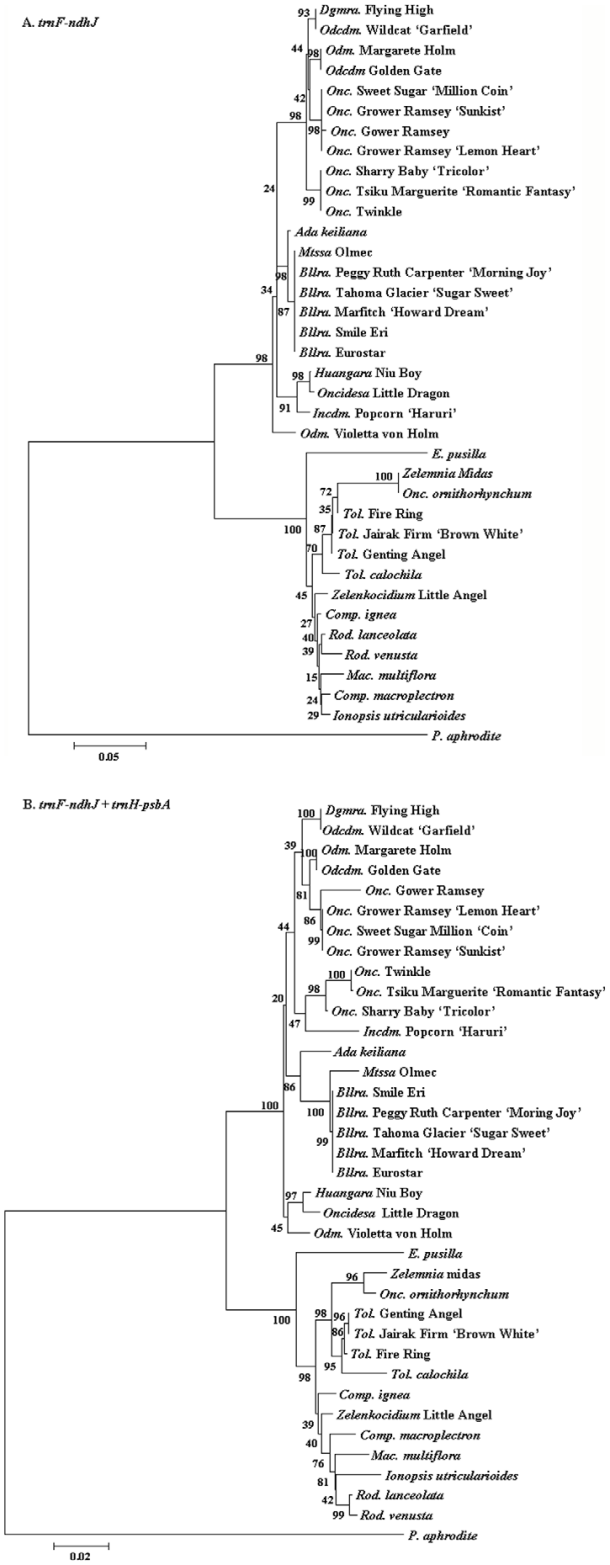
Phylogenetic analysis of 36 Oncidiinae species. These trees are based on the nucleotide sequences of A. *trnF*-*ndhJ*, B. *trnF*-*ndhJ* and *trnH*-*psbA* cpDNA regions. The numbers indicate bootstrap probability values. The names of genera are abbreviated as follows: *Bllra*., *Beallara*; *Comp*., *Comparettia*; *Dgmra*., *Degarmoara*; *Incdm*., *Ionocidium*; *Mac*., *Macradenia*; *Mtssa*., *Miltassia*; *Odm*., *Odontoglossum*; *Odcdm*., *Odontocidium*; *Onc*., *Oncidium*; *P*., *Phalaenopsis*; , *Rod*., *Rodriguezia*; and *Tol*., *Tolumnia*.

With the exception of members of the *Miltassia* and *Beallara* genera, other species could be separated well using *trnF*-*ndhJ* region, although the bootstrap scores were low (Figure 5A). We therefore combined 2 regions, *trnF*-*ndhJ* and *trnH*-*psbA*, for phylogenetic analysis (Figure 5B). The combined analysis gave a similar, but more distinguishable, result when compared with that using the *trnF*-*ndhJ* region alone. The *Miltassia* genus was separated from *Beallara*. *Onc*. *ornithorhynchum* and other *Oncidium* species were separated into 2 different groups. Phylogenetic analysis showed that *E. pusilla*, *Rodriguezia*, and *Tolumnia* were grouped together, distinct from the *Oncidium*, *Odontocidium* and *Beallara* group.

## Discussion

### Advantages of next generation sequencing and BAC libraries for chloroplast genome sequencing

In Taiwan, analysis of orchid genomic sequence have provided valuable information for investigating molecular mechanisms of orchid flowering development, perspectives, and disease resistance pathway [4], [19]–[21]. In the other hand, total cp genomes are useful for evolutionary studies [28]. Total DNA or chloroplast DNA had been used on several occasions as basic materials to obtain cp genomes [8], [40]–[42], but in such studies the possibility of DNA contamination could not be entirely ruled out [43], [44]. Other studies show that DNA fragments can be transferred between chloroplasts, mitochondria, and nuclear genomes during evolution [45]–[47]. Sequences of mitochondria and chloroplasts of rice and maize share high percentage sequence homology [48], [49]. There are 68 kB cpDNA sequences (42.4% of the cp genome) in the mtDNA of *V. vinifera* [50]. To reduce the possibility of DNA contamination in our study, we applied BAC library screening by using chloroplast genes as probes for sequencing the complete chloroplast genomes.

For cp genome sequencing, a shotgun library [31] and a PCR-based method were used [9], [51]. The PCR-based method relies on the sequence conservation of the chloroplast genome. The products were further validated with Sanger sequencing. Recently, NGS has become a powerful tool for genome sequencing as it is time-saving, low in cost and uses high-throughput technology [40], [52]. Various chloroplast genomes, such as 6 woody bamboos belonging to the BEP clade with controversial internal relationships [52] have been successfully sequenced by NGS. However, using NGS sequencing and a BAC library has not previously been used to sequence a complete orchid cp genome.

*E. pusilla* cp genome BAC clones were identified in our study by using PCR screening. By using chloroplast specific primers [9], the BAC library could be screened using PCR [10], which is easier and faster than traditional hybridization methods [53]. Meanwhile, we also identified BAC plasmids with mitochondria clones which contained chloroplast homologous sequences. Several single nucleotide polymorphisms (SNP), insertion/deletion and homology sequences were found between the mitochondria and chloroplast sequences in *E. pusilla* (data not shown). Using Illumina sequencing and chloroplast specific BAC plasmids, the possibility of reassembly errors caused by homologous sequences between chloroplast and mitochondria or nucleus DNA could be excluded. Combining a BAC library screened using a PCR approach and Illumina sequencing, we obtained an accurate chloroplast genome sequence efficiently and economically. The chloroplast genome studies will support the identificationy of phylogenitically close relatives of *Erycina* and will help in their breeding and genetic improvement.

### Characteristics of *E. pusilla* chloroplast genome

The gene order of the 3 orchid chloroplast genomes was very similar. Unlike other monocot plants, such as maize, rice, and wheat (NC_001320, NC005973, NC_002762, NC_001666), the 3 orchid cp genomes contained the *ycf2* gene, which is similar to dicot plants, such as tobacco, *Arabidopsis*, and Lotus (NC_001879, NC_000932, NC_002694) [8]. The 153 bp longer *ycf2* in *P. aphrodite* (Figure 2) is also found in other dicot plants. The function of the short 153 bp *ycf2* in *E. pusilla* and *Onc*. Gower Ramsey needs further exploration.

The products of the *ndh* genes catalyze the transfer of electrons from NADH to plastoquinine, which adjusts the redox level of the photosynthetic electron transporters [54]. Most *ndh* genes in the chloroplast genome of *E. pusilla*, *Onc*. Gower Ramsey, *P. aphrodite* and the other 14 Oncidiinae species were deleted or truncated [8], [9]. The non-functional *ndh* genes are also found in crassulacean acid metabolism (CAM) and C3 plants, such as *Pinus thunbergii*, *Ketekeeria davidiana*, *Ephedra equisetina* and *Welwitshia mirabilis*, which belong to autotrophic, heterotrophic, genmospermae, or monocot species, respectively [55]–[57]. Furthermore, in *Erodium* genus, 11 plastid-encoded *ndh* genes were intact in *Ero*. *texamum* and *Ero*. *carvifolium*, but were deleted in *Ero*. *chrysnthum*. No morphologic or biological features are associated with *ndh* gene loss in *Erodium* [58]. These results indicate that the loss-of-function of chloroplast encodeing the *ndh* genes might not affect photosynthesis. The ancestral plastid *ndh* genes of orchids are presumed to have been transferred to the nucleus [8]. The orchid nuclear genome sequences, which are still unavailable, are needed to clarify the horizontal gene transfer questions of *ndh* genes in orchids.

### Analysis of 5 regions in 19 Epidendroideae species

In the *petN*-*psbM* region, 3 species belonging to the Collabiinae shared a deletion of the same size (Table 2, and at the same position, Figure 4B). However, a 20 bp longer deletion was found in *Geo*. *densiflorum* and *Pha*. *mishmensis*, which belong to the Eulophiinae and Collabiinae subtribes, respectively. Therefore, the *petN*-*psbM* deletion found in the three Collabiinae species might not specific to the Collabiinae subtribe.

Four species belongs to Cymbidieae tribe including *E. pusilla*, *Onc*. Gower Ramsey, *Cym*. *aloifolium* and *Geo*. *Densiflorum* shared the same deletion in the *rps15*-*trnN* region that is located between the SSC and the IR (Table 3, Figure 3E). .SSC/IR is one of the most variable loci and could be an evolution marker [59]. To examine whether this deletion is commonly exits in Cymbidieae tribe, SSC to IR sequences of Epidendroideae were download from the NCBI database and further analyzed [60], [61]. The deletion in *rps15*-*trnN* was conserved in all 77 Cymbidieae tribe, including species belonging to Oncidiinae, Crytropodiinae, Eulophiinae, and Maxillarieae subtribes (Figure S1). Similar to *P. aphrodite*, other species belonging to the Vandeae tribe, Podochileae tribe, Collabiinae subtribe, or Dendrobieae subtribe contained no such deletion. Together, these findings indicate the deletions in the *rps15-trnN* were commonly exits in Cymbidieae tribe.

### Phylogenetic analysis of Oncidiinae species

Due to the unique morphology, *E. pusilla* has rendered taxonomic classification and denomination a continual challenge [62]. In 2001, *E. pusilla* was finally named according to the molecular systematics which were performed based on the *ITS*, *matK*, and *trnL*-*F* DNA regions [63]. The fast-growing feature of *E. pusilla* makes it a good parent for breeding. To supply molecular information for *Oncidium* breeding, 36 Oncidiinae species including tropical-adaption commercial hybrids were further analysis. Though the *matK* region was a good marker for Oncidiinae phylogeny investigation [63]. In our study, the *matK* regions within 36 Oncidiinae commercial species were too conserved to separate well. The *matK* regions might not a good marker to make phylogenetic inferences within commercial hybrids. Another primer for the IRb-SSC region failed to produce a PCR product for many species, which also made phylogenetic analysis using a 4 DNA region combination difficult in this study. However, the phylogenetic analysis using the two regions *trnF*-*ndhJ* and *trnH*-*psbA* were able to demonstrate a good resolution within 36 Oncidiinae including commercial hybrids. *E. pusilla* is located close to the *Tolumnia* and *Rodriguezia*, while *Ionocidium*, *Oncidesa*, and *Oncidium* belong to another group (Figure 4). The phylogenetic result is able to explicate the hybridization compatibility of *E. pusill*a [27]. No fruit set and seed production in *Ionocidium* Popcorn ‘Haruri’ and *Oncidesa* Little Dragon crossed with *E. pusilla*. Fruit can be obtained by crossing *E. pusilla* with *Rod*. *lanceolata* but only few seed would be germinated. However, fruits and progeny could be germinated successfully by crossing *E. pusilla* with several species belonging to the *Tolumnia* genus including *Tol*. Genting Angel. Our phylogenetic analysis using *trnF*-*ndhJ* and *trnH*-*psbA* thus provides a reference for the hybridization compatibility of *E. pusilla*. For traditional hybridization breeding, this is important information to select new hybrid parents systematically and create new commercial species efficiently.

Previously, orchid taxonomy has been based on floral traits and morphological features. However, classification is changed frequently because the characteristics of orchid are easily affected by interspecific or intergeneric crossing and changes in environment. *E. pusilla* and *Zelenkocidium* Little Angel used to be taxonomically grouped into the *Oncidium* because of their similar floral appearances. Currently, molecular taxonomy has started to reveal more precise phylogenetic relationship and many species of orchid have been renamed and reclassified. For example, *Onc*. Little Angel was reclassified as *Zelenkocidium* Little Angel. *Onc*. Midas, the hybrid of *Zelenkoa onusta* and *Oncidium flexuosum*, was renamed as *Zelemnia* Midas. According our results, *Zelemnia* and *Zelenkocidium* are located close to *Tolumnia* and distant from *Oncidium* in the phylogenetic tree in the current study (Figure 4) thus validating previous results. Beyond the species that we have examined, there might be other species that should be moved to *Zelemnia* or *Zelenkocidium* from *Oncidium* despite having a similar floral appearance to *Oncidium*. The phylogenetic tree showed *Odontoglossum* Violetta was phylogenetically distant from *Odontoglossum* and *Odontocidium*, and *Onc*. *orithorhynchum* was much closer to *Erycina* and *Tolumnia* than *Oncidium* and *Odontoglossum*. We therefore suggest that the taxonomy of *Odm*. Violetta and *Onc*. *orithorhynchum* should be further checked and compared with the parent. Similar misplacements might occur in many commercial *Oncidium* species. a possibility that requires further investigation. Accurate Orchid taxonomy is not only desirable for evolutionary studies, but is important for orchid breeding.

## Supporting Information

Figure S1 Sequences of Oncidiinae (yellow), Cyrtopodiinae (red), Eulophiinae (cyan), and Maxillarieae subtribe (pink) of Cymbidieae tribe, Vandeae tribe (green), Podochileae tribe (orange), Sobralieae subtribe (blue), Collabiinae subtribe (gray), and Dendrobieae subtribe (purple) were downloaded from NCBI and analyzed by using VectorNTI AlignX software program.
(TIF)
