# Supplementary figures and images for: Complete Chloroplast Genome Sequence of an Orchid Model Plant Candidate: Erycina pusilla Apply in Tropical Oncidium Breeding
Source: PLoS One. 2012 Apr 4;7(4):e34738. doi: 10.1371/journal.pone.0034738 (PMC3319614; doi:10.1371/journal.pone.0034738)

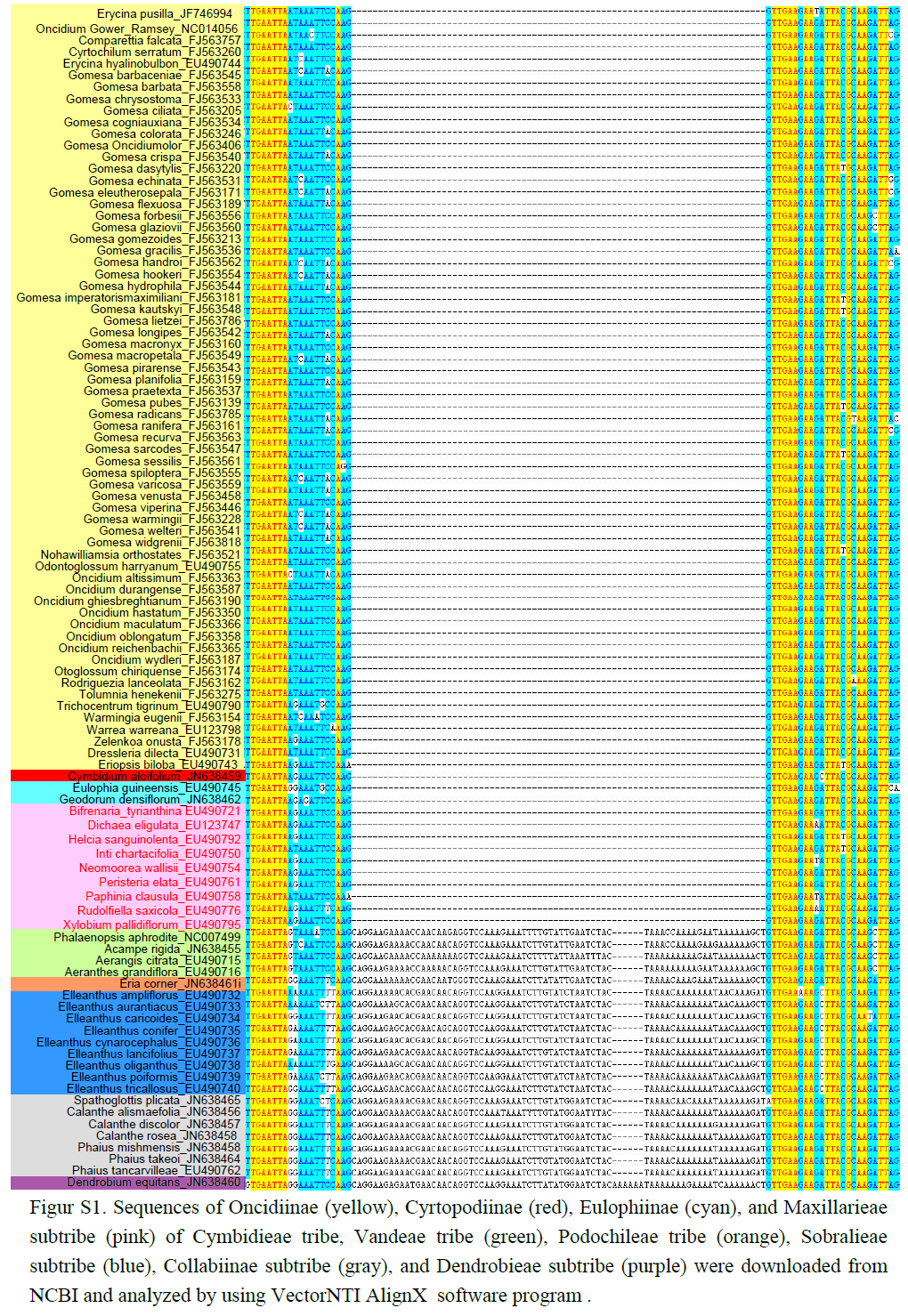

Supplement: Figure S1 — Sequences of Oncidiinae (yellow), Cyrtopodiinae (red), Eulophiinae (cyan), and Maxillarieae subtribe (pink) of Cymbidieae tribe, Vandeae tribe (green), Podochileae tribe (orange), Sobralieae subtribe (blue), Collabiinae subtribe (gray), and Dendrobieae subtribe (purple) were downloaded from NCBI and analyzed by using VectorNTI AlignX software program. (TIF) [file pone.0034738.s001.tif]
